# New derivatives of the antimalarial drug Pyrimethamine in the control of melanoma tumor growth: an in vitro and in vivo study

**DOI:** 10.1186/s13046-016-0409-9

**Published:** 2016-09-06

**Authors:** Chiara Tommasino, Lucrezia Gambardella, Maria Buoncervello, Roger J. Griffin, Bernard T. Golding, Manuela Alberton, Daniele Macchia, Massimo Spada, Bruna Cerbelli, Giulia d’Amati, Walter Malorni, Lucia Gabriele, Anna Maria Giammarioli

**Affiliations:** 1 Department of Therapeutic Research and Medicine Evaluation, Section of Cell Aging and Degeneration, Istituto Superiore di Sanita, 00161 Rome, Italy; 2 Newcastle Cancer Centre, Northern Institute for Cancer Research, School of Chemistry, Bedson Building, Newcastle University, Newcastle upon Tyne, NE1 7RU UK; 3 Department of Hematology, Oncology and Molecular Medicine, Istituto Superiore di Sanità, Rome, Italy; 4 Department of Radiological, Oncological and Pathological Sciences, Sapienza University of Rome, Policlinico Umberto I, Rome, Italy

**Keywords:** Antimalarial Drugs, Chemotherapy, Antifolates, Apoptosis, Melanoma, Drug repurposing

## Abstract

**Background:**

The antimalarial drug Pyrimethamine has been suggested to exert an antitumor activity by inducing apoptotic cell death in cancer cells, including metastatic melanoma cells. However, the dose of Pyrimethamine to be considered as an anticancer agent appears to be significantly higher than the maximum dose used as an antiprotozoal drug.

**Methods:**

Hence, a series of Pyrimethamine analogs has been synthesized and screened for their apoptosis induction in two cultured metastatic melanoma cell lines. One of these analogs, the Methylbenzoprim, was further analyzed to evaluate cell-cycle and the mechanisms of cell death. The effects of Methylbenzoprim were also analyzed in a severe combined immunodeficiency (SCID)-mouse xenotransplantation model.

**Results:**

Low dose of Methylbenzoprim was capable of inducing cytotoxic activity and a potent growth-inhibitory effect by arresting cell cycle in S-phase in melanoma cells. Methylbenzoprim was also detected as powerful antineoplastic agents in SCID-mouse although used at very low dose and as a single agent.

**Conclusions:**

Our screening approach led to the identification of a “low cost” newly synthesized drug (methylbenzoprim), which is able to act as an antineoplastic agent in vitro and in vivo, inhibiting melanoma tumor growth at very low concentrations.

**Electronic supplementary material:**

The online version of this article (doi:10.1186/s13046-016-0409-9) contains supplementary material, which is available to authorized users.

## Background

Metastatic melanoma has a poor prognosis and frequently develops resistance to standard therapies. Recently, new treatment options have been evaluated in the clinic and the results with immunotherapy and targeted therapy were promising [1]. Nevertheless, the initial excitement about the possibility of having discovered new effective approaches to treat melanoma has been followed by a degree of discouragement because these therapies are usually associated with high costs, side effects and none appears to be curative when used as a single agent [2, 3]. Their efficacy may be enhanced in combination with other chemotherapeutic agents [4, 5]. Actually, the drug dacarbazine, and its orally active analogue temozolomide (TMZ) remain the gold standard in melanoma chemotherapy. However, the response rate of melanoma to these drugs is approximately 20 %, underscoring the need to develop more effective treatments [6].

In this context, recent evidence has shown that antimalarial drugs could be beneficial in the treatment of different types of tumors [7, 8]. Pyrimethamine (2,4-diamino-5-p-chlorophenyl-6-ethyl-pyrimidine; Pyr) is already used in humans as an orally administered drug for the treatment of infections caused by protozoan parasites (e.g. malaria and toxoplasma). Pyr belongs to the group of antifolate drugs inhibiting dihydrofolate reductase (DHFR), an enzyme, which is essential for the synthesis of folic acid, a cofactor for DNA synthesis [9]. DHFR inhibitors, e.g. methotrexate, have been studied for many years as anticancer agents since antifolates have greater selective toxicity toward rapidly dividing cells such as tumor cells. Previous studies have also demonstrated that Pyr is a potent pro-apoptotic inducer in cancer cells, e.g. in metastatic melanoma cells [10, 11]. It has been suggested that the mechanism underlying this activity involves both the activation of the caspase cascade (e.g. caspases 8–9 and 3) and cathepsin cascade (e.g. cathepsin B). A remarkable inhibition of cell growth and a S-phase cell cycle arrest was also demonstrated.

Unfortunately, dose-related adverse effects of Pyr have been described since its introduction in the clinical practice, including bone marrow suppression with leukopenia, thrombocytopenia and megaloblastic anemia [12–15]. Although, some experimental studies suggested that a dose of 50 mg/day of Pyr could be well tolerated [16, 17], clinical trials have also shown that unwanted effects are connected to the individual variability of patients. On these bases, Pyr should be used with caution in patients with impaired immune system, heart diseases and reduced renal or hepatic function. In regard to this, different studies have shown that the highest risks are found in individuals treated with Pyr at or above 50 mg/day [15, 18–23]. According to the above described potential toxicity and taking into consideration the therapeutic potential of Pyr, as well as the demand for drug repositioning of low-cost agents such as Pyr [24, 25], a series of Pyr derivatives has been synthesized and screened for their activity on melanoma. We report here the results obtained in melanoma cultured cells and in xenografted animals that point at one of these Pyr-analogs, the MBP, as capable of hindering metastatic melanoma tumor growth at concentrations 10-folds lower than Pyr.

## Methods

### Cell lines

Human metastatic melanoma cell lines MeWo and Mel501 were cultured in RPMI 1640 (Life Technologies, Invitrogen) supplemented with 10 % heat-inactivated fetal bovine serum (Euroclone), 2 mmol/L glutamine (Sigma-Aldrich, St. Louis, MO, USA) and 50 μg/mL gentamicin (Sigma). Tumor cells were tested as Mycoplasma-free (Mycoplasma detection kit; (Roche, Switzerland).

### Synthesis of Pyrimethamine analogues

**Fig. 1:**
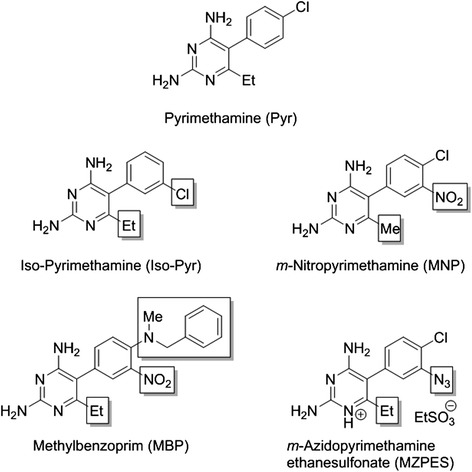
Chemical structures. Structures of Pyrimethamine (Pyr), Iso-Pyrimethamine (Iso-Pyr), *m*-Nitropyrimethamine (MNP), Methylbenzoprim (MBP) and *m*-Azidopyrimethamine ethanesulfonate salt (MZPES). Structural differences in Pyrimethamine analogues are highlighted by boxes

The target analogues were readily prepared from Pyrimethamine (Pyr) [26, 27]. Briefly, direct nitration of Pyr under standard condition afforded m-nitropyrimethamine (MNP) in near quantitative yield. Reduction of MNP to the corresponding amine, followed by diazotisation and treatment with sodium azide, gave m-azidopyrimethamine (MZP). Conversion to the ethanesulfonate salt (MZPES) was achieved on treatment of MZP with aqueous ethanesulfonic acid. Methylbenzoprim (MBP) was synthesized from MNP in high yield by displacement of the 4-chloro substituent with *N*-benzylmethylamine employing 2-ethoxyethanol as solvent. Iso-Pyrimethamine (Iso-Pyr) was synthesised by following the method of Russell and Hitchings [28]. Structural features of Pyr-analogs are shown in (Fig. 1).

### Chemicals and drugs

Pyr (Sigma), Pyr-analogues indicated above and TMZ (Sigma) were dissolved in DMSO (no precipitation of drug was observed on addition of the DMSO solution to aqueous medium); Leucovorin (Sigma) and Chloroquine (CQ) (Sigma) were dissolved in water and were diluted in RPMI 1640 immediately before experiments. The cathepsin B inhibitor CA-074-Me (Calbiochem, Millipore, Germany) and pan-caspase inhibitor z-VAD-fmk (R&D System, USA) were diluted in RPMI 1640 immediately before experiments.

### Treatments

Melanoma cells were exposed to Pyr (8 μg/ml), TMZ (100 μmol/L), Pyr-analogues (0.8, 8, 80 μg/ml) for 24, 48 and 72 h. In the combined treatments, Leucovorin (LV) (10 μg/ml) was given at the same time points of the above drugs; CQ (10 μM) was given 16 h before the treatment with Pyr or MBP; the cathepsin B inhibitor (CA-074-Me, 10 μmol/L) and the pan-caspase inhibitor z-VAD-fmk (50 μmol/L) were added to the culture medium 2 h before the treatment.

### Cell viability assay

CellTiter 96 AQueous One Solution Cell Proliferation Assay (Promega, Madison, WI) was used to determine cytotoxic effects resulting from treatment with drugs. Melanoma cells were plated at a density of 3 × 10^3^ cells/well in 96-well plates, and then treated with various concentrations of MBP and Pyr for 24, 48, and 72 h. At the end of the treatment period, 20 μl of 3-(4,5-dimethylthiazol-2-yl)-5-(3-carboxymethoxyphenyl)-2-(4-sulfophenyl)-2*H*-tetrazolium (MTS) reagent were added to each well. The plates were incubated for 30 min at 37 °C in the dark. Absorbance at 450 nm was determined on Opsys MR spectrophotometer (DYNEX Technologies, Denkendorf, Germany) using Windows Revelation QuickLink software. Each experimental condition was performed in triplicate and repeated at least twice. All values were normalized with respect to the viability of untreated cells.

### Cell death evaluation

Quantitative evaluation of apoptosis was performed by a double staining flow cytometry method using FITC-conjugated Annexin V (AV)/propidium iodide (PI) apoptosis detection kit (Marine Biological Laboratory, MBL, USA) according to the manufacturer’s protocol. Reported data refer to both early (AV^+^/PI^−^ cells, still alive) and late (AV^+^/PI^+^ cells, dead cells) apoptotic melanoma cells.

### Western blotting analysis

Cell lines were lysed in RIPA buffer (100 mM Tris–HCL pH 8, 150 mM NaCl, 1 % Triton X-100, 1 mM MgCl, 25 mM NaVO_4_) in the presence of complete protease-inhibitor mixture (Sigma). Immunoblotting was performed with antibodies to: caspase-9 (rabbit polyclonal antibody, Enzo Life Sciences); caspase-8 (mouse monoclonal antibody, Transduction Laboratories); caspase-3 (mouse monoclonal antibody, Enzo Life Sciences); cathepsin B (rabbit polyclonal antibody, Calbiochem). As a control, the membranes were incubated with specific antibodies of anti-α-tubulin (mouse monoclonal antibody, Sigma). The intensities of bands of active fragments were quantified normalizing to Tubulin. The optical density of the bands [integrated area in arbitrary units (AU)] was measured by using the National Institutes of Health Image J software (rsb.info.nih.gov/ij).

### Cathepsin B

Anti-cathepsin B polyclonal antibody (Calbiochem) was used to evaluate the expression of activated cathepsin B by flow cytometry analysis. Control and treated cells were fixed with 4 % paraformaldehyde in phosphate-buffered saline (PBS) for 30 min at room temperature. After washing in the same buffer, cells were permeabilized with 0.5 Triton X-100 (Sigma) in PBS for 5 min. Then cells were incubated with Anti-cathepsin B polyclonal antibody (Calbiochem) and after 30 min at 37 °C, cells were washed and then incubated with anti-rabbit fluorescein-linked whole antibodies (Molecular Probes, Eugene, OR, USA).

### Caspase activity

Caspase-9, caspase-8 and caspase-3 activities were assayed by using the CaspGLOW fluorescein active caspase staining kit (MBL), following the manufacturer’s instruction, as previously described [18]. Western blot was performed as described above.

### Cell cycle analysis

Cultured cells were treated with 1 mmol/L bromodeoxyuridine (BrdU; BD Immunocytometry Systems) for 30 min, removed from culture and fixed in 70 % ice-cold ethanol. 1 × 10^6^ fixed cells were incubated in 3 N HCl for 20 min. After washing with 0.1 mol/L Na_2_B_4_O_7_ (pH 8.5) to stop acid denaturation, cells were washed twice with 1 % bovine serum albumin and 0.5 % Tween 20 and labeled with an anti-BrdU FITC-conjugated (BD Immunocytometry Systems) for 30 min at 4 °C. Cells were then stained with 40 μg/mL PI (Sigma) in the presence of 10 μmol/L RNase (Sigma) for 30 min at 37 °C followed by analysis on a flow cytometer.

### Analysis of autophagy

Detection of autophagy was performed by using Cyto-ID Autophagy Detection Kit (Enzo Life Sciences, Lausen Switzerland). The kit was optimized for detection of autophagy in live cells by flow cytometry. CYTO-ID® Autophagy Detection Kit measures autophagic vacuoles and monitors autophagic flux in lysosomally inhibited live cells using a dye that selectively labels accumulated autophagic vacuoles [29]. The probe is a cationic amphiphilic tracer (CAT) dye that rapidly partitions into cells in a similar manner as drugs that induce phospholipidosis. Careful selection of titratable functional moieties on the dye prevents its accumulation within lysosomes, but enables labeling of vacuoles associated with the autophagy pathway. Induction of autophagic flux can be visualized by enhanced accumulation of autophagic vesicles if lysosomal function is inhibited, preventing removal of these vesicles.

This assay provides a rapid, specific and quantitative approach for monitoring autophagic activity at the cellular level by using a 488 nm-excitable probe that becomes fluorescent in vesicles produced during autophagy. Western blot was also performed, as described above.

### Animals

CB.17 SCID/SCID female mice (Harlan Italy) were used at 4 to 5 weeks of age and were kept under specific pathogen-free conditions as previously described [11]. Before injection of Mycoplasma-free melanoma cells, mice were weighed and divided into two control groups (untreated and DMSO-treated) and two treatment groups (Pyr and MBP) with eight mice per group. Mice were injected s.c. into the right flank with 2 × 10^6^ melanoma Mel501 cells per mouse. Suspensions of Pyr (45 mg/kg) were prepared daily in 5 % of DMSO in aqueous methylcellulose solution and the suspensions of MBP (12 mg/Kg) in 5 % of DMSO. At the onset of tumor (i.e. 5 d from melanoma cell injection), mice were given by oral gavage with the same volume (200 μL) of vehicle or Pyr (45 mg/kg) or MBP (12 mg/Kg) 5 d per week up to 40 d from injection (35 d of drug administration). All mice were inspected daily and the overall clinical condition was assessed. Tumor growth was monitored by measuring maximal and minimal diameters by caliper and tumor weight was estimated with the formula: tumor weight (mg) = [length(mm) × width^2^(mm)]/2. Procedures and facilities followed the requirements of Commission Directive 86/609/EEC concerning the protection of animals used for experimental and other scientific purposes. Italian legislation is defined in D.L. no. 116 of January 27, 1992.

### Histological analysis

Livers were formalin-fixed and paraffin embedded. Consecutive sections (5–6 μm thick) were stained with hematoxylin-eosin and observed under a light microscope (Olympus Corporation of the Americas, Center Valley, USA).

### Statistical analyses

All samples were analyzed with a FACSCalibur cytometer (BD, Biosciences, Heidelberg, Germany) equipped with a 488 argon laser. At least 20,000 events were acquired. Data were recorded by a Macintosh computer using CellQuestPro Software (BD). Statistical analyses were performed by using Student’s *t*-test for paired samples or non-parametric Anova test. All data reported were verified in at least three different experiments and reported as mean ± S.D. *P*-values < 0.05 were considered as statistically significant.

## Results

### Pyrimethamine analogues induce apoptosis in human melanoma cell lines

**Fig. 2:**
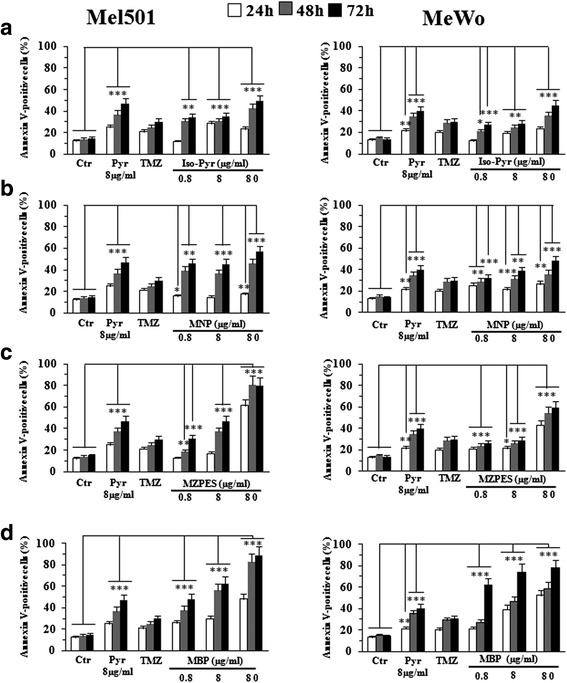
Proapoptotic effects of Pyr-analogues versus Pyrimethamine and temozolomide on Mel501 and MeWo cell lines. Flow cytometric analysis of apoptosis after the treatment with Pyr (8 μg/ml), TMZ (100 μmol/L) and Pyr-analogues (0.8, 8, 80 μg/ml) for 24, 48 and 72 h on Mel501 (left panels) and MeWo (right panels) cell lines. *Columns*, mean values of 10 independent experiments; bars, SD. *, *P* < 0.05; **, *P* < 0.01, *** *P* < 0.001 significance compared with untreated cells. All screened compounds, Iso-Pyr (**a**), MNP (**b**), MZPES (**c**) and MBP (**d**), were able to induce apoptosis but only one of these, MBP (**d**), was more potent than Pyr and TMZ even at very low concentration (0.8 μg/ml) in both cell lines. Note the significant difference between MBP-treated and TMZ-treated samples after 48 and 72 h even at lower concentration of MBP (0.8 μg/ml)

It has already been shown that Pyr is a potent apoptosis inducer in cancer cells [8–11, 17]. Thus, we first evaluated the apoptosis-inducing potential of Pyr-analogues in two human metastatic melanoma cell lines (MeWo and Mel501) by using annexin V/propidium iodide (AV/PI) double staining assay. Parallel analyses were carried out in cells treated with Pyr and the chemotherapeutic agent TMZ at a clinically relevant concentration (100 μmol/L) [30]. We tested Pyr-analogues using the same dosage of Pyr (8 μg/ml), which is known to induce cell death in melanoma cells [10, 11] at different time points, i.e. after 24, 48 and 72 h of treatment. In addition, lower and higher doses (10-fold lower or higher) were also evaluated. In Fig. 2, we reported the percentage of annexin V-positive cells (apoptotic cells) detected in the two cell lines. All screened compounds were able to induce apoptosis, but only one of these was more potent than Pyr and TMZ. In particular, the compound MBP was able to induce apoptosis even at 10-fold lower concentration (0.8 μg/ml) in both cell lines (Fig. 2d, left and right panels). Of note, for all tested concentrations, the percentage of necrotic cells (AV-/ PI+ cells) was below 4 % with the exception of the higher dose of MBP (80 μg/ml), which induced high levels of PI single positive cells (necrotic cells) in both cell lines at all time points tested (Table 1). On the basis of the screening reported above, the MBP compound was selected for further, more detailed analyses using the 0.8 and 8 μg/ml doses.

**Table 1:** Single-color staining with Propidium iodide

Mel501	Ctr	Pyr	Temozolomide	MBP 0.8ug/ml	MBP 8ug/ml	MBP 80ug/ml
24 h	0.4	1	0.1	0.6	2	11.5
48 h	1.2	4	4	2	3.6	14
72 h	1	3.6	3	2.4	4.2	25
MeWo	Ctr	Pyr	Temozolomide	MBP 0.8ug/ml	MBP 8ug/ml	MBP 80ug/ml
24 h	0.5	1	0.8	0.9	0.9	9.5
48 h	0.8	0.5	1	1.6	1.4	15
72 h	1	0.7	0,8	1	1	18

Numbers in the columns represent the average percentage of AV negative/PI positive (necrotic cells) obtained by 10 different experiments

### MTS cell viability assay and determination of the IC_50_ of Methylbenzoprim

**Fig. 3:**
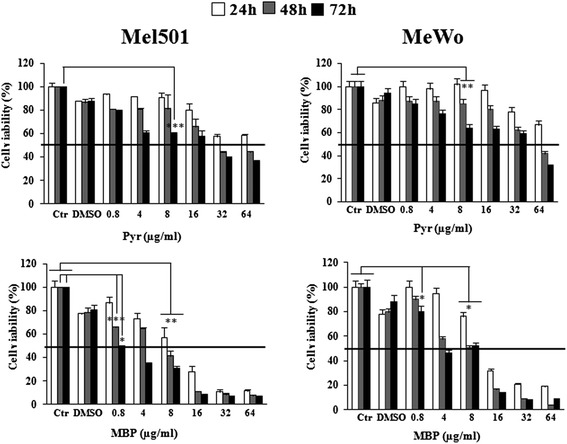
Antiproliferative effects of Methylbenzoprim and Pyrimethamine on Mel501 and MeWo cell lines. Viability of drug-treated Mel501 (*left*) and MeWo (*right*) cell lines was evaluated at 24, 48 and 72 h by MTS assay. Each value is normalized to untreated cells. Data are the means ± S.D. of three independent experiments bars, SD. *, *P* < 0.05; **, *P* < 0.01, *** *P* < 0.001 significance compared with untreated cells. We observed a time- and dose-dependent decrease of cell proliferation in Pyr and MBP treated cells, but at different doses (from 0.8 to 64 μg/ml). In particular, after 72 h of exposure Mel501 showed an IC_50_ of 0.8 μg/ml for MBP, indicating a much higher activity of this agent with respect to Pyr, characterized by an IC_50_ ranging between 16 and 32 μg/ml. Similarly, MeWo cells resulted in much more susceptibility to the antiproliferative effects of MBP, since the IC_50_ of this agent ranged between 4 and 8 μg/ml as compared with that of Pyr (32–64 μg/ml)

Since the newly synthesized compound MBP appears to be effective at lower doses than Pyr, we evaluated it in vitro IC_50_ value versus that of Pyr by MTS assay in both Mel501 and MeWo cell lines. Treatment of the two human melanoma cell lines was performed for 24, 48 and 72 h at concentrations ranging between 0.8 and 64 μg/ml. As shown in Fig. 3, a significant higher suppression of cell growth by MBP versus Pyr was observed in both types of cancer cells. Although for both drugs we observed a time and dose-dependent inhibition of cell proliferation, this event occurred earlier and at lower doses when cells were treated with MBP. In particular, MBP showed higher cytotoxic potential against Mel501 cells with IC_50_ = 0.8 μg/ml after 72 h of exposure, with respect to Pyr, characterized by IC_50_ ranging between 16 and 32 μg/ml. Similarly, MeWo cells resulted in much more susceptibility to the anti-proliferative effects of MBP, since, after 72 h, the IC_50_ of this agent ranged between 4 and 8 μg/ml as compared with that of Pyr (32–64 μg/ml).

### Methylbenzoprim induces cathepsin B and caspase dependent apoptosis on melanoma cells

**Fig. 4:**
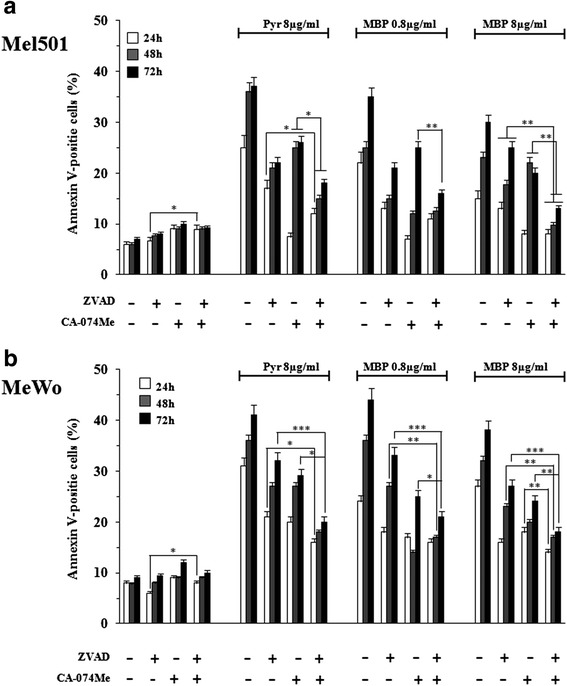
Pan-caspase inhibitor Z-VAD-FMK and the cathepsin B inhibitor CA-074-Me inhibit Methylbenzoprim-induced apoptosis in melanoma cells. The percentage of apoptotic cells was evaluated after 24, 48 and 72 h of MBP (0.8, 8 μg/ml), the positive control Pyr (8 μg/ml) and untreated cells in Mel501 (panel **a**) and MeWo (panel **b**) cell lines pretreated for 2 h with the pan-caspase inhibitor Z-VAD-FMK (50 μmol/L) or the cathepsin B inhibitor CA-074-Me (10 μmol/L) or both. *Columns*, mean values of three independent experiments; bars, SD. *, *P* < 0.05; **, *P* < 0.01, *** *P* < 0.001 significance compared with Pyr and MBP treated cells. Z-VAD-FMK induced a protective effect in a time dependent manner in Mel501 and MeWo cell lines (panels **a** and **b**, respectively). When cathepsin B inhibitor CA-074-Me was used, apoptosis was significantly abrogated after 24 h treatment; this effect persisted after 48 h, while it was less evident after 72 h. Z-VAD-FMK and CA-074-Me, administered simultaneously, increased protective effect in both cell lines at all time points analyzed (panels **a** and **b**)

In previous studies we showed Pyr induced apoptosis in melanoma cells via a mechanism that brought into play both the caspase and cathepsin cascades [11]. To identify the apoptotic pathway being activated in response to MBP (the caspase, the cathepsin pathway or both), Mel501 and MeWo cell lines were initially pre-treated with: i) the pan-caspase inhibitor Z-VAD-FMK and ii) the cathepsin B inhibitor CA-074-Me, at different time points (Fig. 4a and b). Consistent with our previous study [11], pan-caspase (z-VAD-fmk) and cathepsin B (CA-074-Me) inhibitors exerted protective effects at all time points studied in both Mel501 (Fig. 4a) and MeWo (Fig. 4b) cell lines. In particular, z-VAD-fmk induced a protective effect in a time-dependent manner in both cell lines (Fig. 4a and b). Interestingly, the observed effects were equally powerful at both low and high dosages of MBP (0.8, 8 μg/ml). Similarly, when cathepsin B inhibitor CA-074-Me was used (Fig. 4a and b), apoptosis was significantly abrogated after 24 h of treatment and this effect persisted after 48 h but was less evident after 72 h. When caspase and cathepsin B inhibitors were administered simultaneously, cell death appeared much more inhibited in both cell lines at all time points analyzed (Fig. 4a and b).

**Fig. 5:**
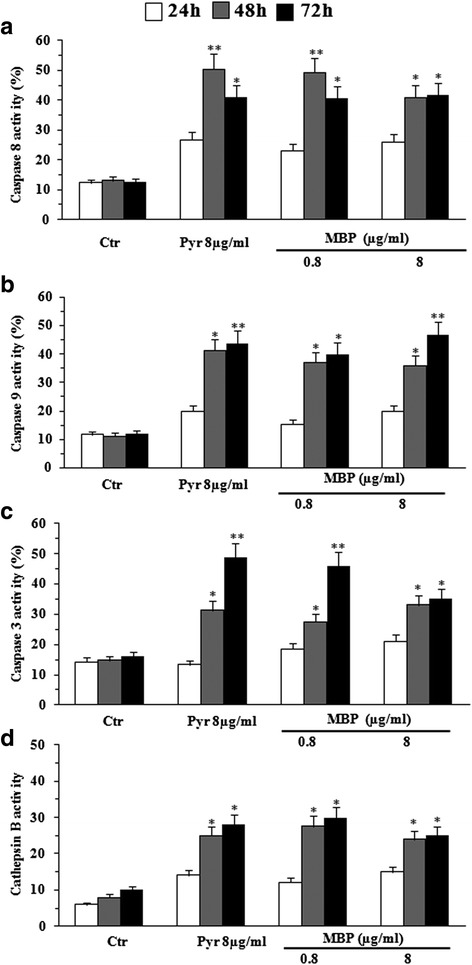
Flow cytometric analysis of apoptotic proteases. The activation of caspases 8, caspase 9, caspase 3 and cathepsin B were evaluated at 24, 48 and 72 h of Pyr (8 μg/ml) and MBP (0.8, 8 μg/ml) treatment on Mel501 cells. Both caspases (8, 9 and 3) and cathepsin B are involved in MBP induced apoptosis. Flow cytometric data showed: i) a significant increase of caspase-8 activity starting from 24 h, reaching a peak at 48 and the plateau at 72 h (**a**); ii) activation of caspase-9 and caspase-3 after 48 and 72 h treatment (**b** and **c**,); iii) a significant increase of expression cathepsin B starting from 48 h, which persisted at later time points (72 h) (**d**), after MBP treatment (0.8 μg/ml). The higher dose of MBP (8 μg/ml) did not induce more relevant effects on the above proteases. Columns, mean values of three independent experiments; bars, SD. *, *P* < 0.05; **, *P* < 0.01; significance compared with untreated cells

**Fig. 6:**
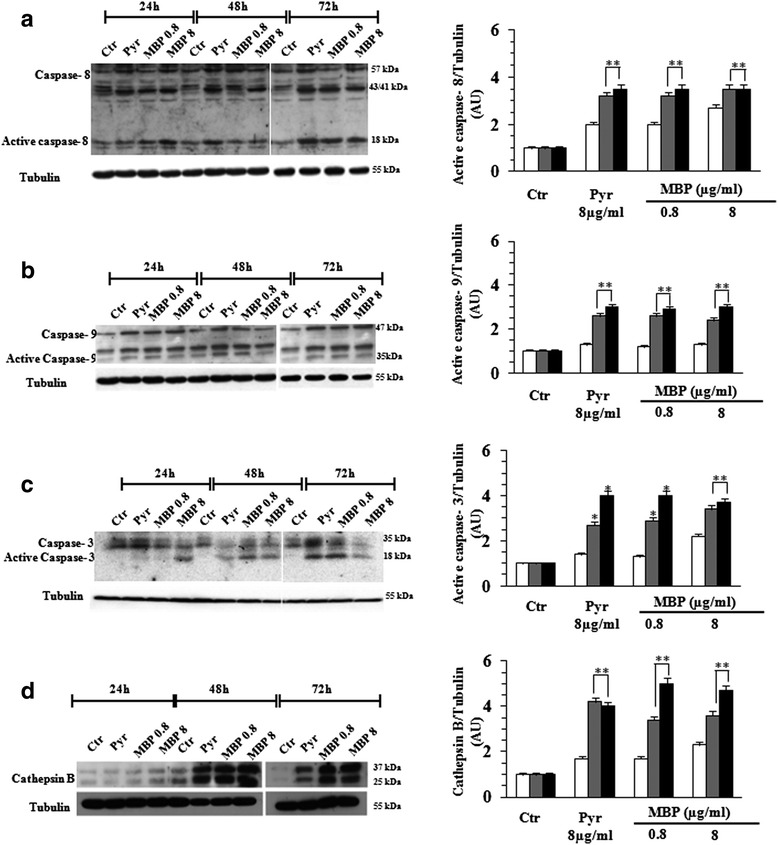
Western Blot analysis of apoptotic proteases. Western Blot analysis confirmed the activation of apoptotic proteases in Mel501 cells treated with Pyr (8 μg/ml) and MBP (0.8, 8 μg/ml). Note that procaspase-8 (57 kDa) is early cleaved into the intermediate forms p43 and p41 and finally processed to the active p18 subunit (Fig. 5a, left panel), and procaspase 9 (47 kDa) is cleaved in 35 kDa subunit (Fig. 5b, left panel). The cleavage of caspase 3 (17 kDa) (Fig. 5c, left panel) and Cathepsin B (25 kDa) (Fig. 5d, left panel) occurred at later time points. The intensities of bands of active caspase fragments were quantified by densitometric analysis (Fig. 6 **a**-**d** right panels). The values were normalized to Tubulin and expressed as arbitrary units (AU). Columns, mean of three independent experiments; bars, SD. *, *P* < 0.05; **, *P* < 0.01

In consideration of overlapping results in the two tested melanoma cell lines, hereafter only data obtained with the Mel501 cell line will be reported. The experiments that follow were performed by comparing the effects induced by both dosages of MBP (0.8, 8 μg/ml) with Pyr (8 μg/ml) (Fig. 5). Hence, in accord with apoptosis inhibition results reported above, we next examined the possible involvement of caspases in the antitumor effects of MBP by both flow cytometric (Fig. 5) and Western blot analyses (Fig. 6): the upstream caspases caspase-8, (mainly involved in receptor-mediated apoptosis) and caspase-9 (mainly involved in mitochondria-mediated apoptosis): caspase-3 (an executioner caspase); the lysosomal protease cathepsin B. The lower dose of MBP (0.8 μg/ml) showed effects comparable to that of Pyr at 8 μg/ml, whereas the higher dose of MBP (8 μg/ml) did not induce more relevant effects. In particular, flow cytometric data showed a significant increase of caspase-8 activity starting from 24 h, reaching a peak at 48 h and the plateau at 72 h (Fig. 5a) whereas the activation of caspase-9 and caspase-3 was detected after treatment for 48 and 72 h (Fig. 5b and c). The representative histograms have been provided as Additional file 1: Figure S.1A and S.1B. Flow cytometric data also showed a significant increase of cathepsin B activity starting from 48 h, which persisted at later time points (72 h) (Fig. 5d). The representative histograms have been provided as Additional file 1: Figure S.2A and S.2B. Western Blot (Fig. 6 left panels) and densitometric analysis (Fig. 6 right panels) confirmed these results by showing an early activation of caspase 8, whereas caspase 9, caspase 3 and Cathepsin B activation occurred at later time points.

Taken together, these experiments suggest that MBP induces pro-apoptotic effects, mediated by both caspase- and cathepsin-dependent pathways, at a significantly lower dose than Pyr (0.8 μg/ml and 8 μg/ml, respectively).

### Methylbenzoprim induces S-phase cell cycle arrest

**Fig. 7:**
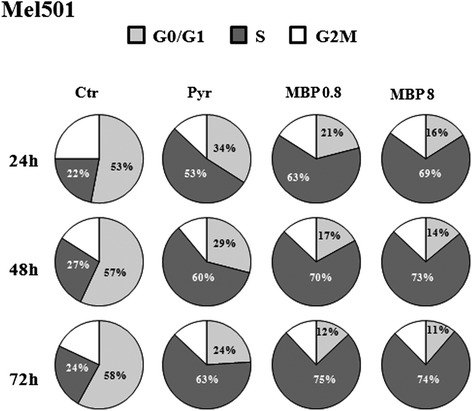
Methylbenzoprim induces S-phase cell cycle arrest. Cell-cycle distribution was evaluated by flow cytometric analysis on Mel501 proliferating cells treated with Pyr (8 μg/ml) and MBP (0.8, 8 μg/ml) for 24, 48 and 72 h. Pie charts show the distribution of cells in the different each phase of the cell cycle: S-phase (*dark grey*), G2/M (*white*) and G0/G1 (*grey*), obtained from three independent experiments. In particular, the low dose of MBP (0.8 μg/ml) was sufficient to induce a high percentage of cells in S-phase as compared to high dose of Pyr (8 μg/ml) at all time points analyzed. The representative histograms have been provided as Additional file 1: Figure S.3

To further investigate the effects induced by low dose of MBP, Mel501 cell cultures were treated with MBP and Pyr as described above and then analyzed for cell cycle distribution. As shown in Fig. 7, both Pyr and MBP hampered cell cycle progression by arresting the cells in S-phase; a corresponding decrease of cells in the G_1_ and G_2_-M phases was also observed. In particular, the low dose of MBP was sufficient to induce a high proportion of cells in S-phase as compared to Pyr (Fig. 7b). The formation of a hypodiploid sub-G1 peak (indicative of cell loss due to apoptosis) was also detected (Additional file 1: Figure S2), confirming the data already evaluated by AV/PI double staining assay (see Fig. 2).

Taken together, these data suggest that a low dose of MBP is able to induce both cell cycle arrest and cell loss due to apoptosis leading to marked antiproliferative effects.

### Dihydrofolate reductase activity

Pyr belongs to the group of antifolate drugs blocking the enzyme dihydrofolate reductase (DHFR) that is essential for the synthesis of folic acid, a cofactor required for DNA synthesis. Previous studies reported that Pyr effects are mainly mediated by its antifolate activity in melanoma cells [10]. To investigate whether the antifolate activity of MBP could contribute to its antiproliferative effects in melanoma cells, we tested the apoptosis-inducing potential of MBP in the Mel501 cell line pre-treated with the reduced form of folate Leucovorin (LV, *N*^5^-formyltetrahydrofolate), a drug widely used to counteract the effects of antifolates [31]. In these experiments, Mel501 cell lines were treated with LV alone and in combination with Pyr (8 μg/ml) and MBP (0.8 μg/ml), after which apoptotic cell death was evaluated.

**Fig. 8:**
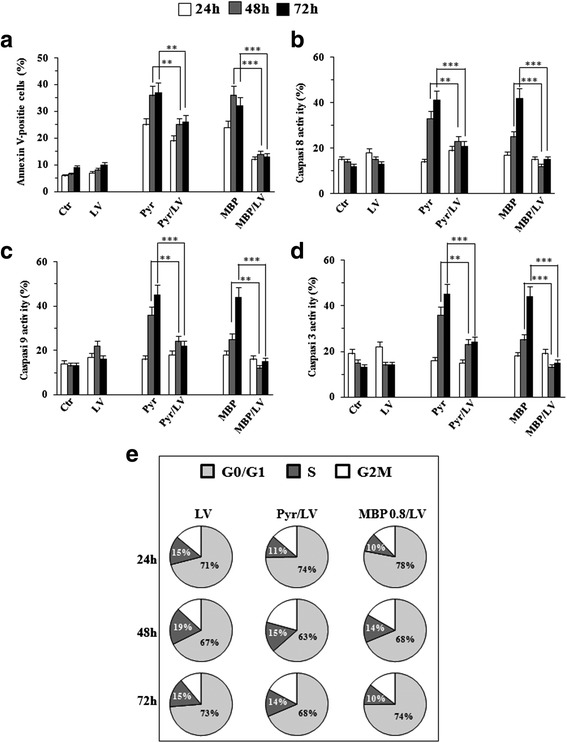
Methylbenzoprim is a potent dihydrofolate reductase inhibitor. Flow cytometric and cell cycle analyses on Mel501 treated with Pyr (8 μg/ml), MBP (0.8 μg/ml) and LV (10 ug/ml) for 24, 48 and 72 h. Flow cytometric data showed: i) LV was only partly able to counteract apoptotic effects induced by Pyr, whereas apoptosis was significantly abrogated at all time points of MBP treatment (**a**); ii) the increased expression of caspase-8 (**b**), caspase-9 (**c**) and caspase-3 (**d**) in both treatments (MBP and Pyr) was significantly reduced in the presence of LV (**b**-**d**) *Columns*, mean values of three independent experiments; *bars*, SD. *, *P* < 0.05; **, *P* < 0.01, *** *P* < 0.001 significance compared with Pyr and MBP treated cells. Pie charts (**e**) show the distribution of cells in each phase of the cell cycle: S-phase (*dark grey*), G2/M (*white*) and G0/G1 (*grey*), obtained from three independent experiments. In particular, LV administration hindered the S-phase cell cycle arrest induced by both Pyr and MBP (**e**)

As shown in Fig. 8a, LV abrogated significantly apoptosis at all time points upon MBP treatment whereas was only partly able to counteract the apoptotic effects induced by Pyr. Since we showed that MBP increased expression of caspases (Fig. 5) and folate deficiency has been suggested to increase the expression of caspases [32], we also analyzed the effects of LV on expression of apoptotic proteases in melanoma cells treated with MBP as compared to Pyr. Interestingly, the increased expression of caspase 8 (Fig. 8b), caspase 9 (Fig. 8c) and caspase 3 (Fig. 8d) induced by both MBP and Pyr treatments was significantly reduced in the presence of LV (Fig. 8b and d). Likewise, LV administration also hindered the S-phase cell cycle arrest induced by both MBP and Pyr (Fig. 8e).

Taken together, these data suggest that the antifolate activity could contribute, at least in part, to the antitumor effects of low dose MBP on melanoma cells, i.e. cell death triggering and cell cycle arrest.

### Autophagy modulation after Pyrimethamine and Methylbenzoprim treatment

**Fig. 9:**
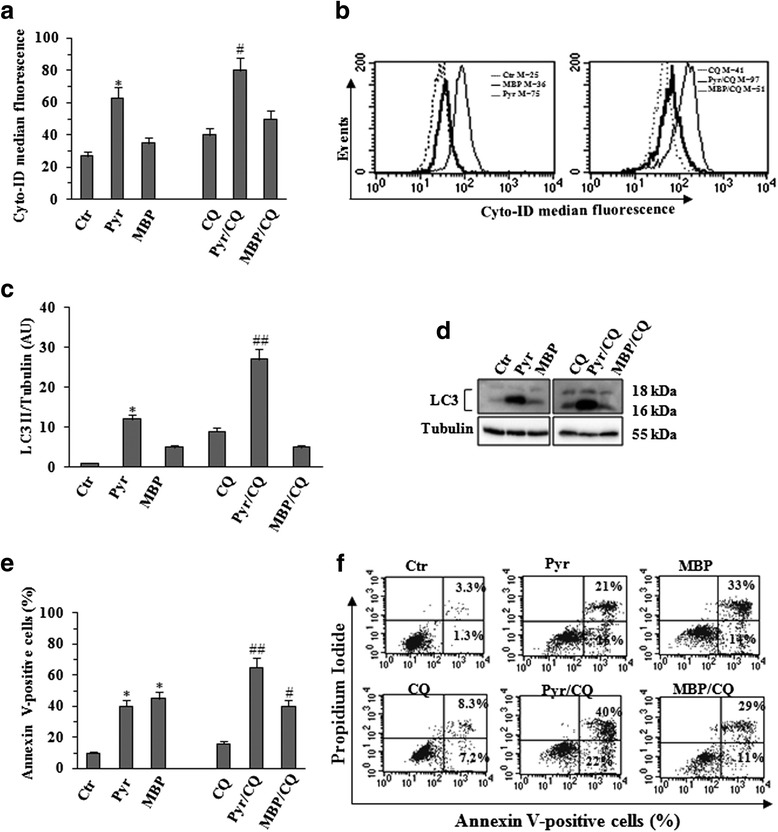
Autophagy modulation after Pyr and MBP treatment. Autophagy was evaluated utilizing two different assays: a cationic amphiphilic dye that selectively labels autophagic vacuoles (Cyto ID autophagy detection kit) by flow cytometric analysis (Fig. 9**a**-**b**) and the expression of LC3I/II by WB analysis (Fig. 9**c**-**d**). Mel501 melanoma cells were treated with Pyr (8 μg/ml), and MBP (0.8 μg/ml) for 48 h. The experiments were performed in the presence or absence of the late autophagy inhibitor CQ (10 μM) for an overnight treatment before the end of the experiment. As shown by fluorescence histograms of Cyto-ID Green autophagy dye (Fig. 9**a**-**b**), Pyr treatment was able to induce a significant increase of autophagic vacuoles. Note, the induction of autophagy can be better visualized when the removal of these vesicles is prevented by CQ. Treatment with MBP did not induce significant autophagy and (as expected) CQ treatment (MBP/CQ) did not induce any significant effect. Figure 9**a**, *Columns*, mean values of three independent experiments; bars, SD. *, *P* < 0.05; significance compared with untreated cells. #, *P* < 0.05; ##, *P* < 0.01; significance compared with CQ treated cells. In Fig. 9**b** the data of a representative experiment were shown. The data were confirmed by western blot of LC3-II turn-over (Fig. 9**d**) in the presence or absence of CQ. The intensities of bands of LC3II were quantified by densitometric analysis (Fig. 9**c**). The values were normalized to Tubulin and expressed as arbitrary units (AU). *Columns*, mean values of three independent experiments; bars, SD. *, *P* < 0.05; significance compared with untreated cells. #, *P* < 0.05; ##, *P* < 0.01; significance compared with CQ treated cells. As Pyr was able to induce both apoptosis and autophagy, we have also evaluated Pyr-induced apoptosis in the presence of CQ. As shown in Fig. 9**e**, CQ significantly enhanced Pyr-induced apoptosis (about 2-fold) while MBP/CQ treatment did not induce any significant effect. *Columns*, mean values of three independent experiments; bars, SD. *, *P* < 0.05; significance compared with untreated cells. #, *P* < 0.05; ##, *P* < 0.01; significance compared with CQ treated cells. In Fig. 9**f** a representative experiment was shown

Available chemotherapeutic agents generally act killing cancer cells by apoptosis whereas drugs inducing autophagy are emerging as able to activate a common pathway of resistance to both standard chemotherapies and novel approaches (e.g. immuno- and targeted therapies) [32]. We have previously reported that autophagy can be modulated by Pyr [33]. Thus, to further compare the effects induced by Pyr and MBP, Mel501 cells were treated with the two antifolate drugs as described above, and then analyzed for autophagy induction. In order to better evaluate the autophagic process, two different assays were used in parallel. First, we used a cationic amphiphilic dye that selectively labels autophagic vacuoles exhibiting bright fluorescence (autophagosome detection by Cyto ID) (Fig. 9a and b) and then we analyzed the LC3-II turn-over by western blot (Fig. 9c and d) The experiments were also performed in the presence or absence of the late autophagy inhibitor CQ, [34] which can be used to examine the autophagic flux. Generally, if autophagy is occurring, the amount of LC3-II as well as autophagic vacuoles will be higher in the presence of the inhibitor. As expected, Pyr alone as well as the combined treatment Pyr/CQ led to an increase of autophagosome detection (Fig. 9a and b) and LC3II expression level (Fig. 9c and d). At variance, according to the data reported above, when we treated melanoma cells with CQ in combination with MBP, no significant effects were found, i.e. no significant changes in autophagosome formation as evaluated by Cyto ID detection kit (Fig. 9a and b) or by evaluating LC3II by western blotting (Fig. 9c and d). As autophagy and apoptosis are two stress-responses that are closely interconnected and Pyr was concomitantly able to induce both apoptosis and autophagy, we have also evaluated Pyr-induced apoptosis in the presence of CQ. Interestingly, the late autophagy inhibitor CQ significantly enhanced Pyr-induced apoptosis (about 2-folds) (Fig. 9e). In Fig. 9f a representative experiment is shown. Conversely, low dose of MBP induced a significant increase of apoptotic rate and no effects were observed adding CQ (Fig. 9e and f).

### Methylbenzoprim reduces melanoma growth in a SCID mouse model

**Fig. 10:**
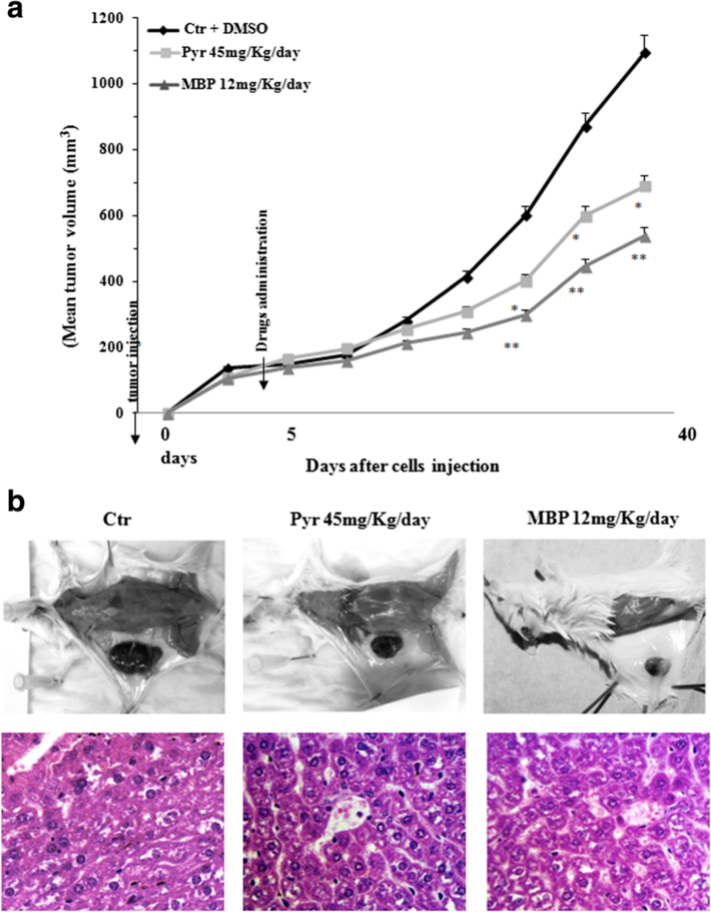
Methylbenzoprim reduces melanoma growth in a SCID mouse model. Tumor growth features in SCID mice inoculated with the human melanoma cell line Mel501 and treated with vehicle alone or Pyr or MBP by oral gavage. Pyr (45 mg/kg/day) and MBP (12 mg/kg/day) treatments were started at the onset of the metastatic tumor (i.e. 5 d after melanoma cell injection). The dose of Pyr used to treat mice was chosen on the basis of previous in vivo studies [11, 24] whereas the dose of MBP was chosen considering both the results of in vitro and preliminary *in vivo* experiments. In (**a**) the mean tumor volume ± SD at different times after melanoma cell injection is reported. Note the significant values detected in comparison with animals treated with vehicle alone (*) = *P* < 0.05; (**) = *P* < 0.01. **b** *Upper panels*. Micrographs show the difference of tumor size between Pyr and MBP, compared with vehicle alone, when mice were sacrificed. Note, in particular, the significant reduction of tumor size observed with MBP compared vehicle alone. *Bottom panels*. Liver histologic features of mice treated with vehicle (*left*), Pyr (*middle*) and MBP (*right*). The liver architecture is well preserved. There is no evidence of hepatocyte necrosis or apoptosis, as compared to the control. Staining with Hematoxylin eosin, original magnification 20×

The in vivo efficacy of MBP was examined by measuring the reduction of tumor growth in a human melanoma xenograft in severe combined immunodeficiency (SCID) mice. Mel501 melanoma cells were injected into the right flank of SCID mice and at the onset of tumor (when tumors had approximately reached 50 mm^3^), mice were treated with Pyr (45 mg/kg) or MBP (12 mg/kg) for 5 days per week up to 40 days from tumor injection for a total 35 days of drug administration. For mice treatments, the doses of Pyr used were selected on the basis of the pharmacokinetic studies carried out in rats [35] and of our previous work performed in SCID mice [11]. In that work, we have analyzed a wide range of Pyr doses (from 3 to 60 mg/ Kg). On the basis of these studies the dose of 45 mg/Kg was considered here [36]. The dose of 45 mg/kg/d corresponds to a plasma concentration of 240 μmol/L [37]. In this study, we have also performed preliminary experiments on SCID mice utilizing a range of doses of MBP from 6 to 45 mg/kg (data not shown). The lowest dose of MBP, which showed antitumor effects comparable to those of Pyr at the dose of 45 mg/Kg, was 12 mg/kg. Of note, the chosen dose of MBP was about three times lower than that of Pyr. Untreated and vehicle treated mice (DMSO) were also included as control groups. Tumor growth was monitored by measuring maximal and minimal diameters by caliper and by evaluating tumor weight, which was estimated as reported elsewhere [11]. In Fig. 10a, the mean of tumor volume at various times after melanoma cell injection is reported. Although a significant reduction of tumor growth (*P* < 0.05) was observed in Pyr treated mice with respect to untreated mice, stronger antitumor effects were obtained with MBP since melanoma grew significantly slower as compared to both vehicle- and Pyr-treatments (Fig. 10a and b). Therefore, MBP exhibits in vivo robust antitumor effects as a lowest dose of this drug than Pyr is sufficient to inhibit more strongly the tumor growth of human melanoma xenograft.

### Comparing In vivo effects of Methylbenzoprim and Pyrimethamine

Tumor growth and treatment with drugs had no effect on the vitality and behavioral responses of animals. No weight loss was observed either during or at the end of the experiment with both treatments. Sacrifice and macroscopic observations of the organs excised from mice at the end of experiments (heart, spleen, kidneys and lungs) showed no macroscopic signs of toxicity in both Pyr and MBP treated groups of mice. Nevertheless, a partially greenish bowel was observed in Pyr-treated mice suggesting a possible occurrence of a blocked bile duct or liver disorders. In order to deepen this aspect, we have further analyzed the liver histologic features of Pyr-treated mice compared to MBP-treated and untreated mice (Fig. 10). As shown in the micrographs (Fig. 10, bottom panels), the liver architecture of Pyr-treated and MBP-treated mice was well preserved and there was no evidence of hepatocyte necrosis or apoptosis.

## Discussion

Successful therapy of metastatic melanoma represents one of the main challenges of chemotherapeutic intervention in the field of cancer control. Although clinical protocols including new biological approaches (e.g. targeted agents, immunotherapy) gave some encouraging results, their efficacy and durable responses remain limited and new evidence indicates their use in combination with chemotherapy [2, 38]. Thus, the search for novel agents capable of exerting anticancer activity appears to be still mandatory. Of great interest, drug repositioning has been growing in importance in the last few years as, by passing much of the early cost and time needed to bring a drug to market, provides a number of low-cost non-cancer drugs for cancer treatment to be exploited in novel anticancer strategies with high therapeutic potential and low-toxicity [39], allowing also access to cures for a higher population of patients. Among these are antimalarials, a class of compounds that have been proposed as anticancer agents thanks to their anti-proliferative activity since 1953 [40]. The reappraisal of one these drugs, Pyr, stems from the encouraging results obtained in the treatment of melanoma and other tumors [7, 8, 10, 11]. In addition, Pyr is already used in humans as an orally administered drug for the treatment of infections caused by protozoan parasites. Of note, Pyr belongs to the group of antifolate drugs that blocks the enzyme dihydrofolate reductase (DHFR). DHFR inhibitors have been studied for many years as anticancer agents for their selective toxicity on rapidly dividing cells such as tumor cells. With this in mind, a series of chemically modified analogues of Pyr has been synthesized and screened in the present work. Here, we report for the first time that one of these, MBP, is a valuable candidate for drug repositioning for cancer treatment as it exerts a powerful effect in both in vitro and in vivo on metastatic melanoma via a mechanism partly overlaying that of Pyr. In particular, multiple effects have been detected: i) apoptosis triggering; ii) activation of cysteine protease, e.g. cathepsin B, activity; iii) inhibition of cell cycle progression, and iv) inhibition of DHFR activity. Of note, MBP activity results associated with the activation of caspase cascade, as either apical caspase (caspase 8–9) or executioner caspase-3 and with the activity of cathepsin B. Interestingly, this lysosomal cysteine protease has been hypothesized for many years as a further actor in the cell death program execution [41–43]. Concerning the cell cycle, the blocking of S-phase and its progression clearly represent an important cytostatic activity of MBP, as for other anticancer drugs [44]. This property could be of great relevance to develop powerful antitumor combination treatments with drugs able to affect cancer cells in S-phase, such as 5-fluorouracil [45]. In this regard, the inhibition of DHFR, probably at the basis of the block in S-phase, could be of relevance since folates are key determinants of cell proliferation and represent essential targets for the control of cancer cell growth, as it appears for the prototypical DHFR inhibitor methotrexate, which nevertheless is endowed with high toxicity [9]. Most importantly, the dose needed for significant in vivo anticancer activity of MBP appears to be about 5-fold lower than that of Pyr, suggesting that lower doses of MBP could show adequate efficacy and cause minor adverse effects compared to the group of antifolate drugs already in use, such as Pyr and, mainly, methotrexate.

A further key point to be considered in anticancer treatments concerns the shift between cell death and cell survival, sustained by apoptosis and autophagy, respectively. Although the role of autophagy in cancer cells is still controversial, recent studies have established that autophagy can be activated to promote the survival of tumor cells when these are exposed to cellular stress conditions such as, radiation, chemotherapy or targeted agents [46]. Autophagy is thus one of the mechanisms that cancer cells have developed to evade therapy-induced cell death. Apoptosis and autophagy are closely intertwined processes and the shift apoptosis/autophagy is extremely delicate and highly dependent on metabolic interactions [33]. Previously, we reported that Pyr (as TMZ and other chemotherapeutic drugs) is able to induce autophagy at the early stage of treatment of tumor cells [33, 47, 48]. In this context, the Food and Drug Administration (FDA) have currently approved the use of CQ in combination with conventional therapies to counteract the mechanisms of cell survival in cancer treatment (source http://www.fda.gov). Therefore, based primarily on the ability to inhibit autophagy, CQ and its derivative, hydroxychloroquine, are currently being investigated as adjuvant therapy in multiple clinical trials for cancer treatment [47], leading to their use as potential chemotherapy and radiotherapy sensitizers rather than antineoplastic. In agreement with these findings, when we combined CQ with Pyr, we found an enhanced cytotoxic effect and block of the autophagic flux. In contrast, in our experiments, MBP did not significantly increase autophagy in comparison with baseline levels found in control samples and its combination with CQ did not modify the apoptotic rate of treated cancer cells. Altogether, our data suggest that Pyr could promotes a limited efficacy in vivo, due to its double-edged activity, which induces both apoptotic cell death and autophagy, therefore, limiting its antitumor activity. Conversely, MBP exerts its strong antitumor activity, probably, by stimulating pro-apoptotic effects only [33]. In addition, as already proposed for antimalarial drug Pyr, the strong efficacy of MBP at very low concentrations suggests that the repositioning of these “low-cost drugs” in cancer chemotherapy could be beneficial.

In a near future, the evaluation of the in vitro and in vivo effects of MBP in combination with new melanoma treatment options appears as mandatory. In particular, further understanding of how MBP could interact with immunotherapy and targeted therapy could present an opportunity for treatment of human metastatic melanoma.

## Conclusion

Recent evidence has shown that antimalarial drugs could be beneficial in the treatment of different types of tumors. Among these the DHFR inhibitor Pyrimethamine (Pyr) is a potent pro-apoptotic inducer in cancer cells. Unfortunately, some dose-related adverse effects have been described since Pyr introduction in clinical practice and this weakened the enthusiasm for its anti-cancer activity.

The results of this study led to the identification of a series of Pyrimethamine analogs that were screened for their ability to induce apoptosis in cultured metastatic melanoma cell lines. All screened compounds were able to induce apoptosis, but only one of these was more potent than Pyr. In particular, the compound Methylbenzoprim (MBP) was able to act as an antineoplastic agent in vitro and in vivo, inhibiting melanoma tumor growth at very low concentrations. Our results suggest that MBP could be considered as a novel agent of interest in the development of new therapeutic strategies against metastatic melanoma.

## Additional file

Additional file 1: Figure S.1A and S.1B. Figure S.1A. Representative image of FACS analysis showing activation of caspase 8, 9 and 3 in Mel501 melanoma cells treated with Pyr and MBP. Figure S.1B. Representative images of FACS analysis showing activation of caspase 8, 9 and 3 in MeWo melanoma cells treated Pyr and MBP. **Figure S.2A**. Representative image of FACS analysis showing activation of cathepsin B in Mel501 melanoma cells treated with Pyr and MBP. **Figure S.2B**. Representative images of FACS analysis showing activation of cathepsin B in MeWo melanoma cells treated with Pyr and MBP. **Figure S.3** Representative images of FACS showing cell cycle analysis on Mel501 melanoma cells treated with Pyr and MBP. Note Pyr and MBP hampered cell cycle progression by arresting the cells in S-phase; corresponding decreases of cells in the G_1_ and G_2_-M phases were also observed. In particular, the low dose of MBP was sufficient to induce a high proportion of S-phase cells, and thereby S-phase arrest, as compared to high dose of Pyr. The formation of a hypodiploid sub-G1 peak (indicative of cell loss due to apoptosis) was also detected confirming the data already evaluated by AV/PI double staining assay. (PPTX 241 kb)

## Abbreviations

AV: Annexin-V
BrdU: Bromodeoxyuridine
BSA: Bovine serum albumin
CA-074-Me: Cathepsin B inhibitor
CQ: Chloroquine
DHFR: Dihydrofolate reductase
ECL: Enhanced chemiluminescence
Iso-Pyr: Iso-Pyrimethamine
LV: Leucovorin
MBP: Methylbenzoprim
MNP: M-nitropyrimethamine
MTS: 3-(4,5-dimethylthiazol-2-yl)-5-(3-carboxymethoxyphenyl)-2-(4-sulfophenyl)-2*H*-tetrazolium
MZP: M-azidopyrimethamine
PBS: Phosphate-buffered saline
PI: Propidium iodide
PVDF: Polyvinylidene fluoride
Pyr: Pyrimethamine
SCID mice: severe combined immunodeficiency mice
SDS-PAGE: Sodium dodecyl sulfate polyacrylamide gel electrophoresis
TMZ: Temozolomide
z-VAD-fmk: Pan-caspase inhibitor
