# Supplementary material for: New derivatives of the antimalarial drug Pyrimethamine in the control of melanoma tumor growth: an in vitro and in vivo study
Source: J Exp Clin Cancer Res. 2016 Sep 6;35(1):137. doi: 10.1186/s13046-016-0409-9 (PMC5013574; doi:10.1186/s13046-016-0409-9)
Supplement: Additional file 1: Figure S.1A and S.1B. — Figure S.1A. Representative image of FACS analysis showing activation of caspase 8, 9 and 3 in Mel501 melanoma cells treated with Pyr and MBP. Figure S.1B. Representative images of FACS analysis showing activation of caspase 8, 9 and 3 in MeWo melanoma cells treated Pyr and MBP. Figure S.2A. Representative image of FACS analysis showing activation of cathepsin B in Mel501 melanoma cells treated with Pyr and MBP. Figure S.2B. Representative images of FACS analysis showing activation of cathepsin B in MeWo melanoma cells treated with Pyr and MBP. Figure S.3 Representative images of FACS showing cell cycle analysis on Mel501 melanoma cells treated with Pyr and MBP. Note Pyr and MBP hampered cell cycle progression by arresting the cells in S-phase; corresponding decreases of cells in the G1 and G2-M phases were also observed. In particular, the low dose of MBP was sufficient to induce a high proportion of S-phase cells, and thereby S-phase arrest, as compared to high dose of Pyr. The formation of a hypodiploid sub-G1 peak (indicative of cell loss due to apoptosis) was also detected confirming the data already evaluated by AV/PI double staining assay. (PPTX 241 kb) [file 13046_2016_409_MOESM1_ESM.pptx]

## Slide 1
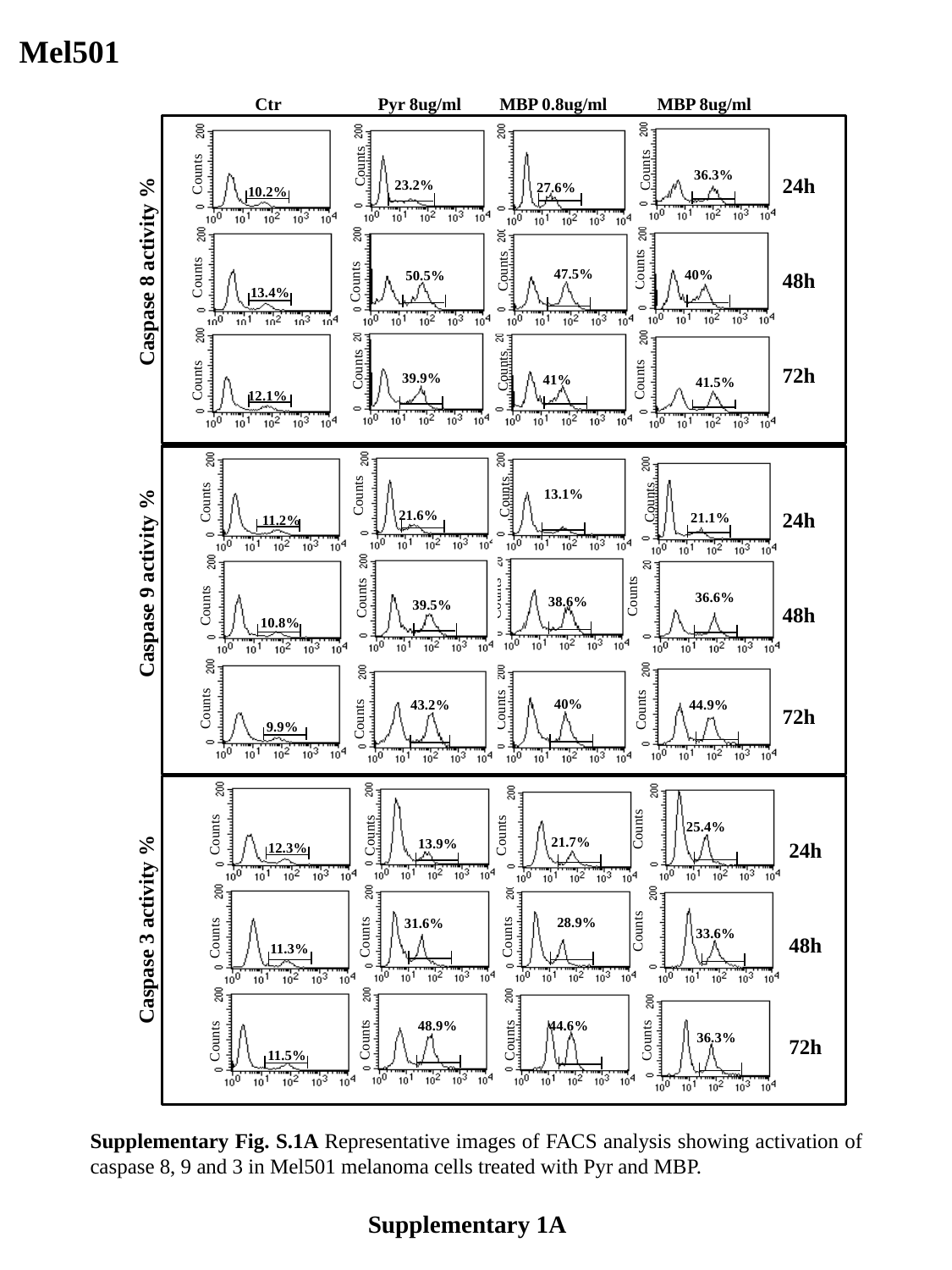

Mel501
Ctr
Pyr 8ug/ml
MBP 0.8ug/ml
MBP 8ug/ml
Counts
36.3%
23.2%
Counts
27.6%
Counts
24h
10.2%
Counts
47.5%
Caspase 8 activity %
Counts
40%
50.5%
48h
Counts
Counts
13.4%
Counts
39.9%
41.5%
Counts
72h
Counts
41%
Counts
12.1%
13.1%
Counts
Counts
21.1%
Counts
Counts
21.6%
24h
11.2%
Counts
39.5%
Caspase 9 activity %
36.6%
Counts
38.6%
Counts
Counts
48h
10.8%
43.2%
Counts
40%
Counts
44.9%
Counts
72h
Counts
9.9%
25.4%
Counts
Counts
Counts
Counts
21.7%
13.9%
24h
12.3%
28.9%
31.6%
Caspase 3 activity %
33.6%
Counts
Counts
Counts
Counts
48h
11.3%
48.9%
Counts
44.6%
36.3%
72h
Counts
Counts
Counts
11.5%
Supplementary Fig. S.1A Representative images of FACS analysis showing activation of caspase 8, 9 and 3 in Mel501 melanoma cells treated with Pyr and MBP.
Supplementary 1A

## Slide 2
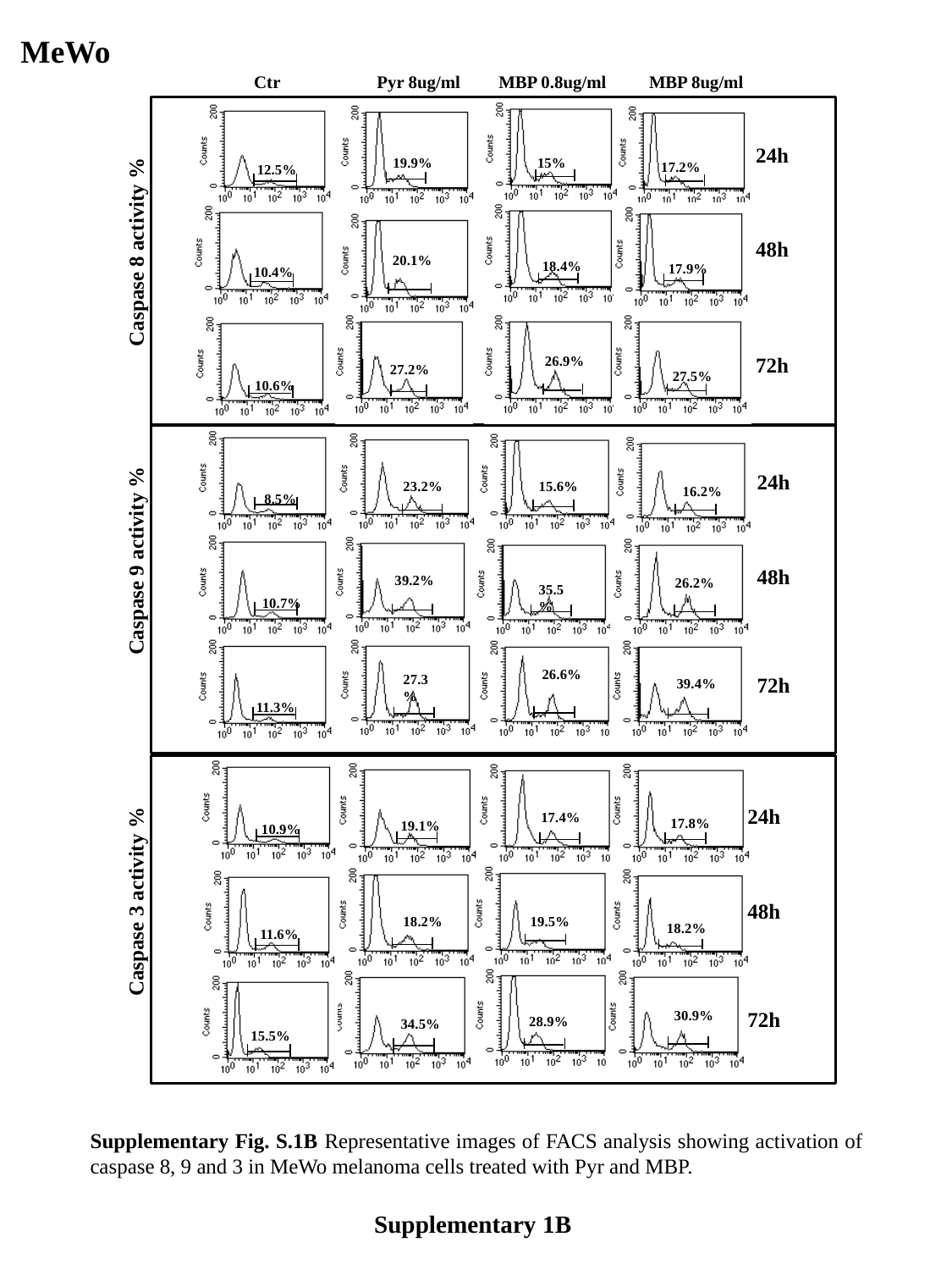

MeWo
Ctr
Pyr 8ug/ml
MBP 0.8ug/ml
MBP 8ug/ml
24h
19.9%
15%
17.2%
12.5%
48h
Caspase 8 activity %
20.1%
18.4%
17.9%
10.4%
72h
26.9%
27.2%
27.5%
10.6%
24h
23.2%
15.6%
16.2%
8.5%
Caspase 9 activity %
48h
39.2%
26.2%
35.5%
10.7%
26.6%
27.3%
72h
39.4%
11.3%
24h
17.4%
17.8%
19.1%
10.9%
Caspase 3 activity %
48h
18.2%
19.5%
18.2%
11.6%
30.9%
72h
28.9%
34.5%
15.5%
Supplementary Fig. S.1B Representative images of FACS analysis showing activation of caspase 8, 9 and 3 in MeWo melanoma cells treated with Pyr and MBP.
Supplementary 1B

## Slide 3
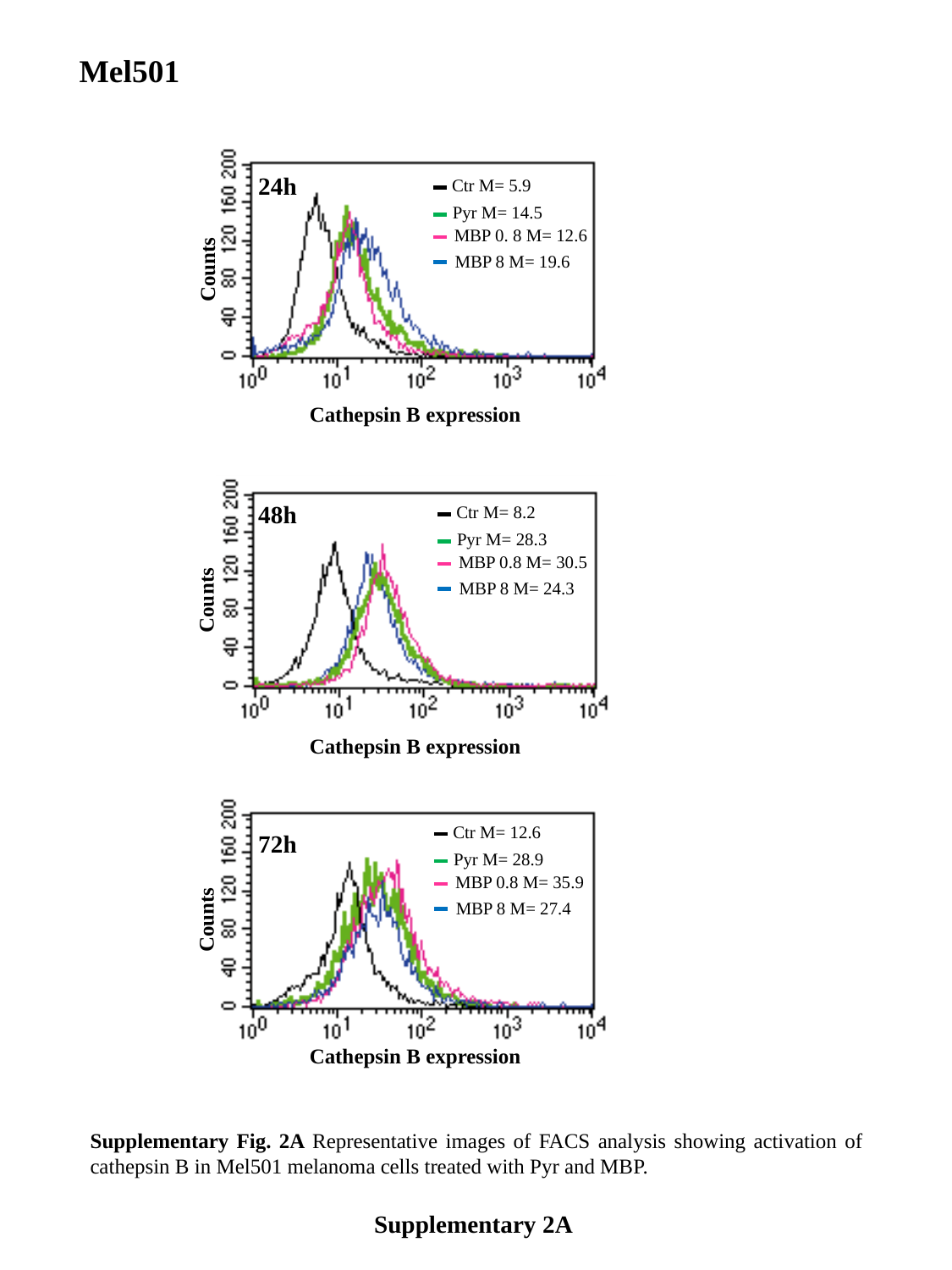

Mel501
24h
Ctr M= 5.9
Pyr M= 14.5
MBP 0. 8 M= 12.6
MBP 8 M= 19.6
Counts
Cathepsin B expression
48h
Ctr M= 8.2
Pyr M= 28.3
MBP 0.8 M= 30.5
MBP 8 M= 24.3
Counts
Cathepsin B expression
Ctr M= 12.6
Pyr M= 28.9
MBP 0.8 M= 35.9
MBP 8 M= 27.4
72h
Counts
Cathepsin B expression
Supplementary Fig. 2A Representative images of FACS analysis showing activation of cathepsin B in Mel501 melanoma cells treated with Pyr and MBP.
Supplementary 2A

## Slide 4
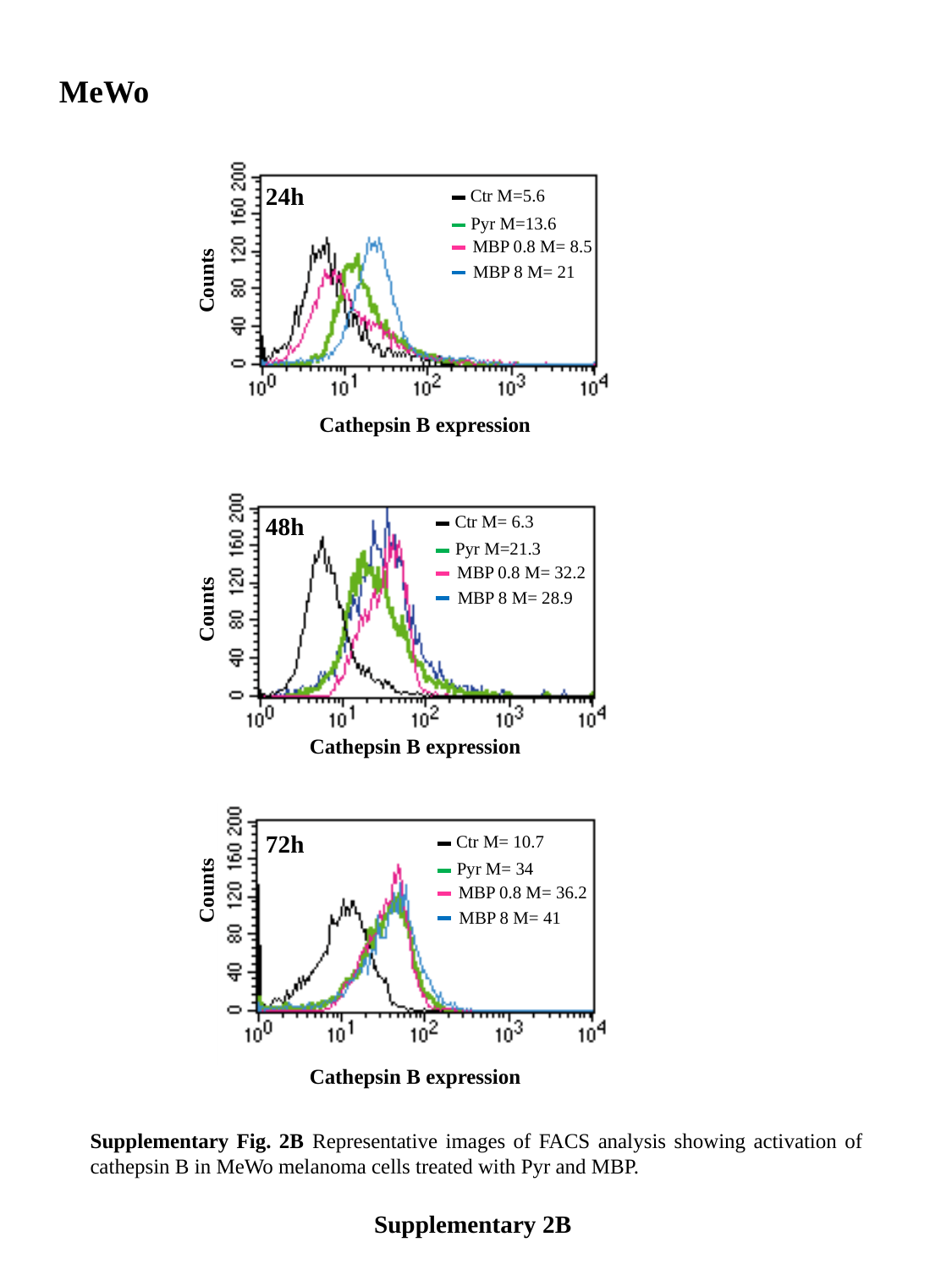

MeWo
24h
Ctr M=5.6
Pyr M=13.6
MBP 0.8 M= 8.5
MBP 8 M= 21
Counts
Cathepsin B expression
Ctr M= 6.3
Pyr M=21.3
MBP 0.8 M= 32.2
MBP 8 M= 28.9
48h
Counts
Cathepsin B expression
72h
Ctr M= 10.7
Pyr M= 34
MBP 0.8 M= 36.2
MBP 8 M= 41
Counts
Cathepsin B expression
Supplementary Fig. 2B Representative images of FACS analysis showing activation of cathepsin B in MeWo melanoma cells treated with Pyr and MBP.
Supplementary 2B

## Slide 5
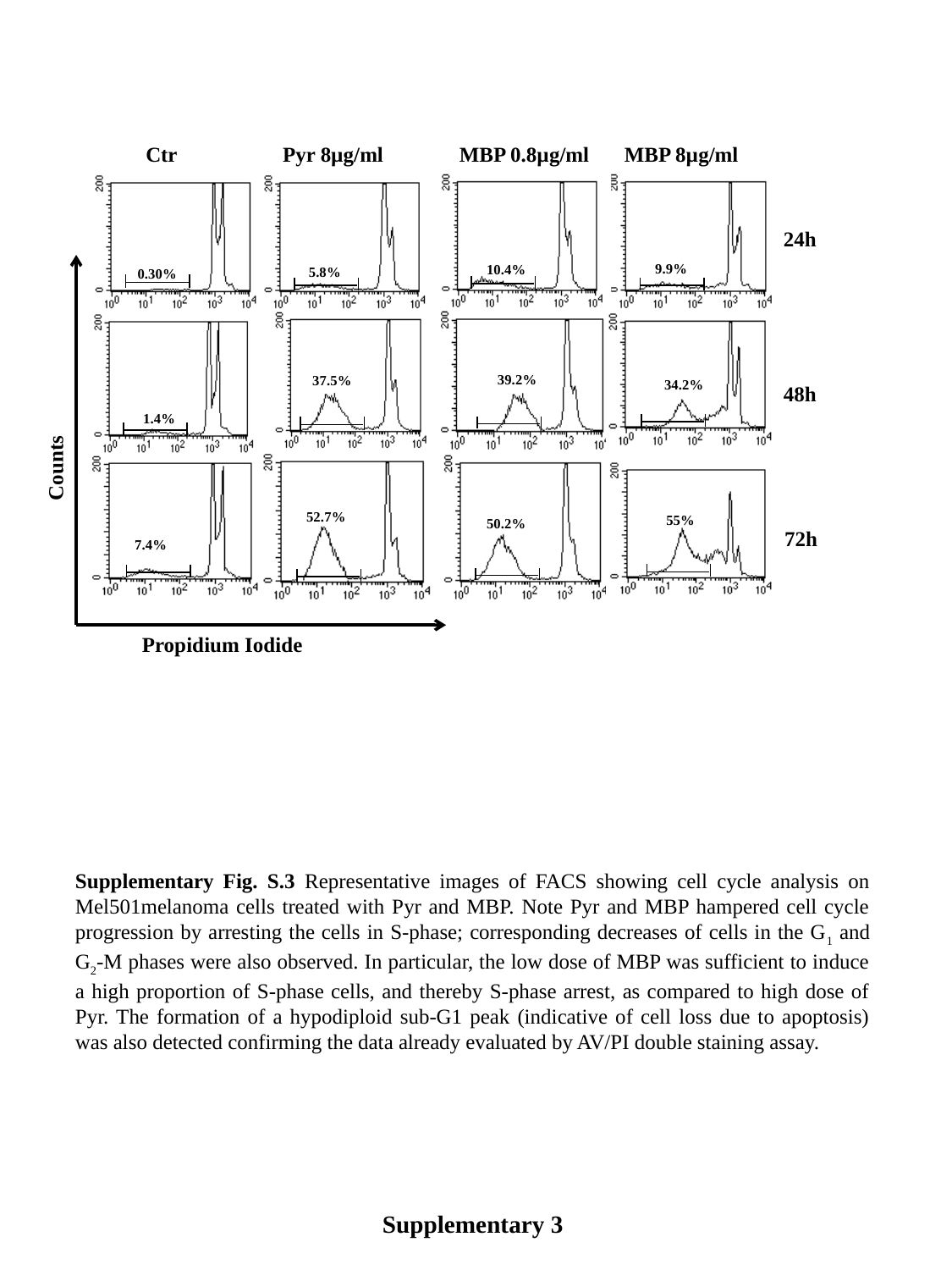

Ctr
Pyr 8µg/ml
MBP 0.8µg/ml
MBP 8µg/ml
24h
9.9%
10.4%
5.8%
0.30%
39.2%
37.5%
34.2%
48h
1.4%
Counts
52.7%
55%
50.2%
72h
7.4%
Propidium Iodide
Supplementary Fig. S.3 Representative images of FACS showing cell cycle analysis on Mel501melanoma cells treated with Pyr and MBP. Note Pyr and MBP hampered cell cycle progression by arresting the cells in S-phase; corresponding decreases of cells in the G1 and G2-M phases were also observed. In particular, the low dose of MBP was sufficient to induce a high proportion of S-phase cells, and thereby S-phase arrest, as compared to high dose of Pyr. The formation of a hypodiploid sub-G1 peak (indicative of cell loss due to apoptosis) was also detected confirming the data already evaluated by AV/PI double staining assay.
Supplementary 3
